# Transdisciplinary research and clinical priorities for better health

**DOI:** 10.1371/journal.pmed.1003699

**Published:** 2021-07-27

**Authors:** Luigi Fontana, Alessio Fasano, Yap Seng Chong, Paolo Vineis, Walter C. Willett

**Affiliations:** 1 Charles Perkins Centre, Faculty of Medicine and Health, University of Sydney, Sydney, New South Wales, Australia; 2 Department of Endocrinology, Royal Prince Alfred Hospital, Sydney, New South Wales, Australia; 3 Department of Clinical and Experimental Sciences, Brescia University Medical School, Brescia, Italy; 4 Division of Pediatric Gastroenterology and Nutrition and Mucosal Immunology and Biology Research Center, Mass General Hospital for Children, Harvard Medical School, Boston, Massachusetts, United States of America; 5 European Biomedical Research Institute of Salerno (EBRIS), Salerno, Italy; 6 Singapore Institute for Clinical Sciences, Agency for Science, Technology and Research (A∗STAR), Singapore; 7 Yong Loo Lin School of Medicine, National University of Singapore, National University Health System, Singapore; 8 MRC Centre for Environment and Health, School of Public Health, Imperial College London, London, United Kingdom; 9 Harvard T. H. Chan School of Public Health, Boston, Massachusetts, United States of America; 10 Channing Division of Network Medicine, Department of Medicine, Brigham and Women’s Hospital and Harvard Medical School, Boston, Massachusetts, United States of America

## Abstract

Modern medicine makes it possible for many people to live with multiple chronic diseases for decades, but this has enormous social, financial, and environmental consequences. Preclinical, epidemiological, and clinical trial data have shown that many of the most common chronic diseases are largely preventable with nutritional and lifestyle interventions that are targeting well-characterized signaling pathways and the symbiotic relationship with our microbiome. Most of the research priorities and spending for health are focused on finding new molecular targets for the development of biotech and pharmaceutical products. Very little is invested in mechanism-based preventive science, medicine, and education. We believe that overly enthusiastic expectations regarding the benefits of pharmacological research for disease treatment have the potential to impact and distort not only medical research and practice but also environmental health and sustainable economic growth. Transitioning from a primarily disease-centered medical system to a balanced preventive and personalized treatment healthcare system is key to reduce social disparities in health and achieve financially sustainable, universal health coverage for all. In this Perspective article, we discuss a range of science-based strategies, policies, and structural reforms to design an entire new disease prevention–centered science, educational, and healthcare system that maximizes both human and environmental health.

## Abstract 2

Luigi Fontana and co-authors discuss present and future challenges, and possible solutions, for global health and health care provision.

Environmental degradation, global warming, and rising pollution are impairing planetary health even as lifestyle- and age-related chronic diseases and emerging infectious diseases are devastating human lives. These are among the greatest challenges facing society today, since people are living longer but often not healthier lives. More than 65% of people over 65 years have 2 or more chronic diseases [1,2]. The current epidemic of obesity, beginning in children, is laying the foundation for even greater problems in the near future, including a reduction in healthy life expectancy. Governmental health expenditure as a percentage of gross domestic product is expected to more than double by 2050, making many existing health funding models unsustainable [3]. Additionally, the present medical approach to chronic diseases in the United States and other affluent countries has vast consequences on planetary health and global economic development. In brief, this reactive “sick-care” medical system is not efficient, equitable, or even viable. Similar problems are now affecting low-income countries, where the epidemiological transition to noncommunicable diseases is coupled with a still high incidence of infectious diseases, dramatic environmental dilapidation, lack of medical resources, and limited support for social and health promotion activities, resulting in increasing inequalities and poverty.

## Lifestyle and prevention of chronic diseases

**Fig 1:**
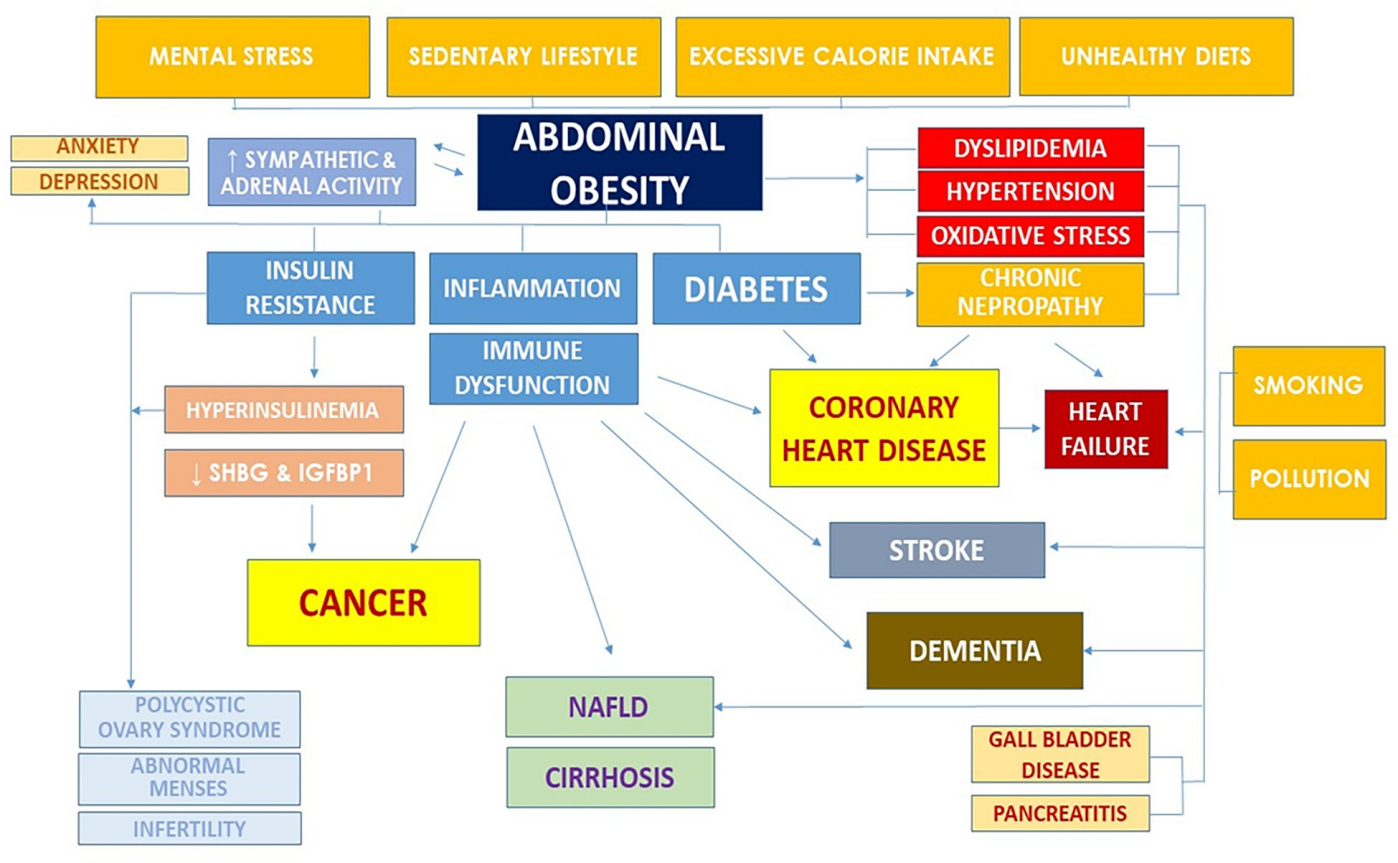
Most prevalent chronic diseases share a common metabolic substrate. The unhealthy lifestyle effectors, including excessive calorie intake, poor diets, sedentary lifestyle, mental stress, smoking, and pollution, modulate important metabolic and hormonal factors associated with the development of the most common age-associated chronic diseases. Abdominal obesity is a central node, but many effects of these unhealthy lifestyle are mediated through other metabolic and inflammatory pathways as well. IGFBP1, insulin-like growth factor-binding protein 1; NAFLD, nonalcoholic fatty liver disease; SHBG, sex hormone–binding globulin.

Thanks in part to extraordinary advances in public health and medicine, life expectancy has more than doubled in the last 150 years, even if this trend is starting to reverse, and the incidence of cardiovascular disease (CVD) and obesity-related cancer is increasing in recent birth cohorts [4–6]. However, many of these chronic diseases are not easily curable because they are multifactorial and are usually controlled with lifelong use of often expensive medications and other devices. Moreover, modern medicine focuses on diagnosing and treating clinically evident diseases one at a time, mainly with drugs and surgery. This approach does not consider that many chronic diseases begin early in life and progress over decades of unhealthy lifestyles, which trigger a wide range of physiologic, metabolic, and molecular alterations, deeply influencing their initiation, progression, prognosis, and therapeutic options (Fig 1).

We believe that universal health coverage should be a right of every human being, but we argue that this is achievable only if we invest adequate resources in preventive science and medicine, and not only in finding new pharmacological targets, an approach that further increases health inequities between rich and poor countries. According to WHO, at least 80% of CVD and diabetes and 40% of cancers are preventable [7]. We believe that these numbers are realistic and probably conservative, because experimental studies have shown that the accumulation of molecular damage can be prevented or much delayed by dietary, genetic, and pharmacological manipulations that down-regulate key cellular nutrient-sensing and inflammatory pathways [8]. In rodents and monkeys, dietary restriction with optimal nutrient intake protects against obesity, diabetes, cancer, CVD, brain aging, and frailty [9,10], and in humans, this induces biological adaptations that protect against those illnesses as well as liver and kidney diseases [11]. Minimizing weight gain during adulthood through regular exercise and a healthy Mediterranean-like diet is key, but specific modulation of other nutritional factors (e.g., specific amino acids, fatty acids, vitamins, and phytochemicals) directly and/or through gut microbiome metabolism may potentiate their beneficial effects [12]. Cognitive training, avoidance of smoking and excessive alcohol consumption, reducing stress, and improving sleep duration and quality are also crucial in preventing harmful physiological alterations [13]. In one study, US men and women who adopted healthier lifestyle behaviors lived about 10 years longer than those who did not, free of major chronic disease [14].

## Intergenerational and life course consequences of preconception and in utero health

The epigenetic theory of disease is another reason why the current medical approach has limited success. Preconception parental environmental factors (diet, fitness, and metabolic and mental health) affect children’s chronic disease risk and influence epigenetic heritage [15]. A high proportion of women experience emotional distress in pregnancy, which seems to be associated with deficits in children’s neurodevelopment [16]. The first 1,000 days of life shaped by maternal diet also influence fetal development, newborn well-being, and health trajectory for the entire life span [17]. This may be in part mediated by shaping the gut, vaginal, and skin microbiome that seems to influence a child’s immunity (and autoimmunity) and cognitive development [18]. Noncommunicable diseases result from multistage processes beginning early in life, but clinical medicine usually intervenes in late stages when therapeutic options are limited and chances of cure are highly decreased.

## Ecological footprint of modern medical systems

The huge progress of medical sciences in treating morbid conditions is undisputed. However, the healthcare sector is responsible for 3% to 10% of CO_2_ emissions, about 10% to 18% of which are due to drug prescription [19]. Moreover, the consumption of old and new medications by a growing number of older adults affected by multiple chronic diseases has caused a 10- to 20-fold increase in aquatic levels of pharmaceutical residues and by-products over the past 20 years, which can now be detected in freshwater and marine organisms, and in crops irrigated with reclaimed water and soil amended with wastewater treatment products [20]. The increasing bioaccumulation and exposure to traces of these pharmaceuticals via food webs, even at low concentrations, raises potential serious concerns for human and environmental health.

## Intensive animal farming and pollution

Diets rich in ultraprocessed and animal foods have deleterious effects not only on humans but also on environmental health. Industrial animal farming account for 70% of freshwater use, with 70% of all land under tillage used to feed livestock. Today, the globalization of agriculture based on extensive production of monoculture crops, together with excessive and inappropriate use of agrochemicals, contribute to deforestation; soil degradation and erosion; groundwater depletion; contamination of rivers, lakes, and aquifers; and release of toxic substances from chemical fertilizers [21,22]. About 15% of greenhouse gas and 20% to 30% of PM10 and PM2.5 in some heavily farmed parts of the world is produced by livestock emissions and by the nitrogen fertilizers [23]. We do not all need to become vegetarians, but substantial movement toward a Mediterranean-like diet, emphasizing whole-plant foods produced with sustainable agriculture practices, would be good for us and the planet [24]. Unfortunately, consumption of meat and dairy is on the rise particularly in low- and middle-income countries [25]. Finally, high-density poultry and swine production creates large reservoirs for the development of antibiotic-resistant bacteria and new viral strains with zoonotic and potential human pandemic effects [26,27]. Although these trends have enormous implications for health, the medical community involvement in mitigation efforts has been limited.

## Benefits of investing in preventive science, education, and medicine

Epidemiological, mechanistic, and translational studies are exponentially elucidating the processes driving the accumulation of organismal damage. Our failure is not due to lack of knowledge, but how we use it. Waiting for millions of people, who eat unhealthy food and engage in harmful lifestyles, to end up in outpatient clinics or hospitals with symptoms of chronic diseases is unethical and financially and environmentally unsustainable. We argue that we should use our accumulating scientific and technological knowledge to increase health equity despite social, economic, and geographical disparities and to minimize the risk of developing diseases, and not only to treat illness after it has clinically occurred.

Transitioning from a primarily disease-centered medical system to a balanced preventive and personalized treatment healthcare system is key. While it is not new to highlight how healthier lifestyles and food systems can address some of these issues, little research and no unifying framework exist to harmonize these concepts of sustainable system management across diverse scientific and medical fields into a coherent theoretical or operational body. Insights beyond reductionist views are needed to encourage integrated changes in the use of our limited financial and human resources, with the aim of achieving their wiser and more productive use.

The real costs of the effects of our dysfunctional medical, food, and agriculture systems, and of our fossil fuel–driven economy, are largely unmeasured and have little or no impact on producers or societal choices about production, distribution, and consumption of goods. Full accounting must become the basis of policy, ethics, and action. This can lead to a range of scientific-based opportunities for cost-effective actions and policies that can be afforded worldwide and add years of healthy life without adding years burdened by disease [14]. Prevention, through the multiple actions we propose in this paper, would have the additional advantage of reducing social disparities in health, now exacerbated by unequal access to education, healthcare, and health-promoting environments [28].

The Coronavirus Disease 2019 (COVID-19) pandemic has also taught us some key lessons that are relevant not only in infection control but also to health in general. First, dealing with emerging diseases requires an open mind, fast insight, scientific rigor, adaptability, and resilience. Second, it demands strong leadership, political restraint, and effective population engagement. Third, it reminds us that health crises exact the greatest toll on the disenfranchised and disadvantaged, both at the individual and country levels. Fourth, it highlights that borders are meaningless in our highly interconnected world and that global solidarity is vital. Finally, we have to learn from the present and the past to develop and implement high-level systems and organizational policies and practices that enhance health literacy. This passage is imperative to move from a doctor-to-patient 1-way model to a continuous 2-way exchange in which the patient takes ownership of his/her health aided by a multidisciplinary healthcare team that facilitates personalized and preventive care.

## Conclusions and future directions

Integrating health literacy as soon as possible into education is key because it shapes health and well-being across people’s lives. However, despite the wealth of mechanistic knowledge linking nutrition, exercise, sleep, and cognitive training and health, these topics receive little or no attention in primary, secondary, and tertiary education, including medical schools. Schools and universities should not be a loose collocation of specialized academic silos but transformative engines that provide not only the expertise needed to have successful careers but also knowledge and practical skills on the mechanisms and interventions linking diet and other lifestyles to human and planetary health.

We need to invest more public and private resources to strengthen existing programs of disease prevention and create more complex and transnational scientific and economic analytic models based on multiple objectives and constraints. Additional resources are needed to develop science-based strategies, effective policies, and structural reforms that encourage integrated pro-health changes, with the aim of achieving better healthcare, diets, and farming systems not only in high-income nations but also in low- and middle-income countries. This includes refining our comprehension of facilitating strategies and barriers to implementation, field testing novel eHealth intervention procedures and materials for efficacy and acceptability by target populations, and improving research and assessment procedures in real-world settings.

Critical global goals, which will require political engagement, should be to develop and deploy evidence-based interventions aimed at reducing behaviors associated with poor health prospects or environmental degradation. These could include, for example, lowering taxes and health insurance premiums for people with healthy lifestyles; making healthy foods more affordable relative to less healthy food, thus reducing health inequalities related to income disparities; taxing not only carbon but also animal and ultraprocessed foods and beverages; ending direct and indirect subsidies for crops fed to animals and intensive animal farming; and restricting advertisement of unhealthy foods to children, implementing front-of-package nutrient warning labels, and enhancing food quality in schools to help curb the growing pandemic of child obesity.

These steps are important individually but take on particular significance when integrated by guiding principles for the design of an entirely new disease prevention–centered science, educational, and healthcare system that maximizes both human and planetary health. Scientific and health organizations should play crucial roles in promoting national and international actions needed to confront challenges to our shared human and environmental health.
